# Epidemiological investigation of fowl adenovirus infections in poultry in China during 2015–2018

**DOI:** 10.1186/s12917-019-1969-7

**Published:** 2019-08-01

**Authors:** Li Chen, Lijuan Yin, Qingfeng Zhou, Peng Peng, Yunping Du, Linlin Liu, Yun Zhang, Chunyi Xue, Yongchang Cao

**Affiliations:** 1 State Key Laboratory of Biocontrol, School of Life Sciences, Sun Yat-sen University, Guangzhou, China; 2 Wen’s Foodstuffs Group Co., Ltd, Yunfu, Guangdong China

**Keywords:** Fowl adenovirus, China, Epidemiology, Phylogenetic analysis

## Abstract

**Background:**

Fowl adenoviruses (FAdVs) are associated with many diseases, resulting in huge economic losses to the poultry industry worldwide. Since 2015, outbreaks of FAdV infections with high mortality rates have been reported in China. A continued surveillance of FAdVs contributes to understand the epidemiology of the viruses.

**Results:**

We isolated 155 FAdV strains from diseased chickens from poultry in China between 2015 and 2018. PCR analysis determined that 123 samples were FAdV species C, 27 were FAdV species E, and five contained two different FAdV strains. The phylogenetic analysis demonstrates that these sequences of hexon regions were clustered into three distinct serotypes: FAdV-4 (79.4%, 123/155), FAdV-8a (13.5%, 21/155) and FAdV-8b (3.9%, 6/155), of which FAdV-4 was the dominant serotype in China.

**Conclusions:**

The characterization of newly prevalent FAdV strains provides valuable information for the development of an effective control strategy for FAdV infections in chickens.

**Electronic supplementary material:**

The online version of this article (10.1186/s12917-019-1969-7) contains supplementary material, which is available to authorized users.

## Background

Fowl adenoviruses (FAdVs) are non-enveloped double-stranded DNA viruses with a genome of 43–45 kb in size and a diameter of 70–100 nm, belonging to the genus *Aviadenovirus* in the family of *Adenoviridae* [1]. The viruses are clustered into 5 species (FAdV-A to FAdV-E) based on restriction enzyme digest patterns, and they are further classified into 12 serotypes (FAdV-1 to 8a and 8b to 11) by cross-neutralization tests [2]. The hexon protein, as a major surface-exposed capsid structure, harbors group, type and subtype-specific antigenic determinants [3, 4]. Therefore, *hexon* gene is important for understanding of the genetic relations of FAdV field strains and the epidemiological status of the virus [5, 6].

FAdVs are disseminated both vertically and horizontally, and widely distributed in poultry industry throughout the world [7–9]. Most of these viruses are opportunistic pathogens and their infections are subclinical [10]. However, some FAdVs are associated with a wide range of avian diseases, including inclusion body hepatitis (IBH), hepatitis-hydropericardium syndrome (HHS) and gizzard erosion ulceration (GEU), causing substantial economic losses to poultry industry [11–13]. Each of the 12 FAdV serotypes has been related to outbreaks of IBH in chickens with 10–30% mortality [5–14]. HHS is primarily caused by the FAdV-4 serotype and causes a high mortality of 30–80% [15]. In addition, FAdV-1 and FAdV-8 strains are responsible for GEU [16, 17]. In recent years, the outbreaks of IBH and HHS showed an increasing trend in poultry industry [18, 19].

In this study, a total of 280 diagnostic samples from chicken flocks were collected from 15 provinces or cities in China from 2015 to 2018. We identified the FAdV strains using sequencing and phylogenetic analysis of the *hexon* genes to establish the genetic relationships of FAdV strains. These results would provide us the information on the epidemiology of FAdV infection, and provide an efficacious control strategy for FAdV infections.

## Methods

### Sample collection and virus isolation

From 2015 to 2018, 280 diagnostic cases were collected from commercial chicken flocks potentially affected by FAdV at ninety-six different chicken farms distributed in 15 provinces or cities in China, including Guangdong, Guangxi, Hunan, Hubei, Jiangxi, Sichuan, Chongqing, Yunnan, Zhejiang, Jiangsu, Henan, Shandong, Hebei, Tianjin and Liaoning. The epidemiological details of the positive isolates are described in Additional file 1: Table S1. Tissue samples (liver, kidney and spleen) were homogenized in phosphate buffered saline (PBS) to obtain a 10% tissue suspension. The suspensions were centrifuged at 5000×g for 10 min after three freeze-thaw cycles. The supernatants were collected, filtered through a 0.22-μm-pore-size syringe filter (Millipore, USA) and inoculated into 7-day-old specific pathogen-free (SPF) chicken embryos which were collected from the SPF Experimental Animal Center (Wens Dahuanong Biotechnology Co., Ltd., Guangdong, China) as previously described [18]. All animals used in this study were unconscious and anaesthesia was maintained with isoflurane in oxygen, and then euthanized by parenteral pentobarbitone at the end of experiment as previously described [20]. Animal experiments were approved by local institutional ethical committee to conduct experiments in chicken embryo and poultry birds as per ethical requirement.

### PCR and sequencing

The presence of FAdV in each sample was confirmed by polymerase chain reaction (PCR), according to procedures described previously [21]. Viral DNAs were extracted from the harvested cultures using the MiniBEST Universal Genomic DNA Extraction Kit (TaKaRa, China), in accordance with the manufacturer’s instructions. Different serotypes of FAdVs were tested by a PCR assay, and typing primers were described in Table 1. The published primers 1–2 were used to amplify the complete *hexon* gene sequences of FAdV-C [19], and the primers 3–4 were used to amplify the complete *hexon* gene sequences of FAdV-E [22]. Samples of the expected length were sent for sequencing directly by Huada Company (Beijing, China).

**Table 1:** Primers used to amplify the complete hexon sequence of FAdV strain

Primers	Sequence (5′-3′)	Products size	Reference
1	F: CGTCTAGGTTCGCACCGCCATGGC	1501 bp	[19]
	R: CATCTGGTCGATGGACCAACGCGCACC		
2	F: CATCGACCAGATGGACAACGTCAACCCCTTCAAC	1345 bp	
	R: TTACACGGCGTTGCCTGTGGCG		
3	F: CGAAGAGGAGACGAAAGC	1942 bp	[22]
	R: TCCCGAGACTGGACTG		
4	F: TACTGCCGTTTCCACATT	1431 bp	
	R: CGGTGTTCACGATAGCC		

### Phylogenetic analysis

The nucleotide sequences of the FAdV isolates were assembled and aligned with homologous sequences via the Clustal W method using the Lasergene software (DNAStar, Madison, WI, USA). A phylogenetic tree for the *hexon* gene was constructed using MEGA 7.0 software by the neighbor-joining method with 1000 bootstrap replicates. 25 reference strains used in this study are summarized in Table 2.

**Table 2:** Fowl adenoviruse (FAdV) reference strains used in the present study

Species	Serotypes	Strains (accession numbers)
A	FAdV-1	CELO(U46933), Phelps (NC001720)
B	FAdV-5	340 (KC493646)
C	FAdV-4	ON1(GU188428), KR5(HE608152), JSJ13(KM096544), HB1510(KU587519), KC (EU177545), SDXT3–15(KU877429), PB-05(EU931691), CG-D (KU647683), MX-SHP95(KP295475), Kr-Yeoju (HQ709228)
	FAdV-10	C-2B(KT717889)
D	FAdV-2	SR48(KT862806), 685(KT862805)
	FAdV-3	SR49(KT862807)
	FAdV-9	A-2A(AF083975)
	FAdV-11	380(KT862812)
E	FAdV-6	CR119(KT862808)
	FAdV-7	YR36(KT862809)
	FAdV-8a	TR59(KT862810)
	FAdV-8b	UPM04217(KU517714), 764(KT862811), HG (GU734104)

## Results

### Epidemiological analysis

**Fig. 1:**
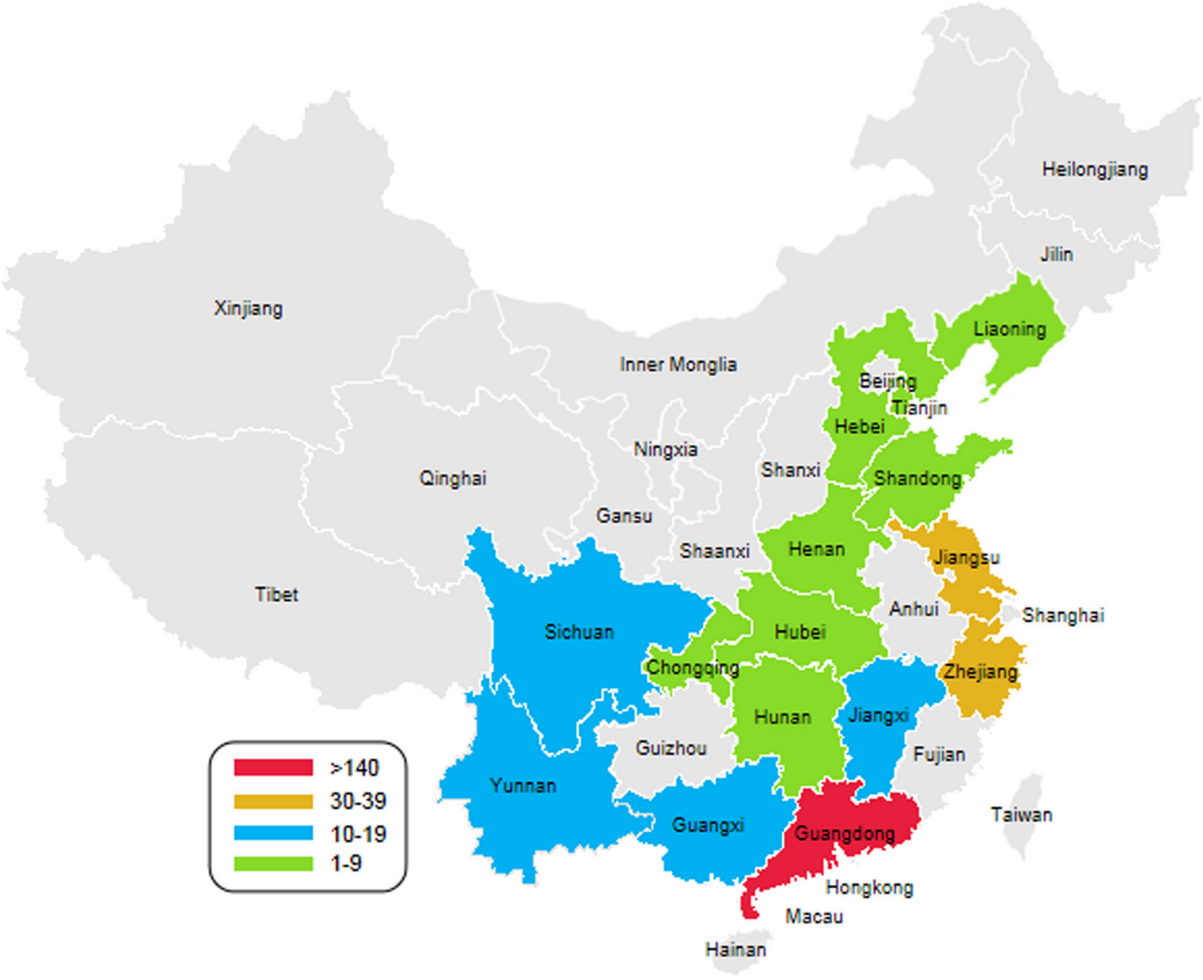
Geographic distribution of collected FAdV samples. The samples were collected from 15 provinces or cities in China. The colours correspond to the number of collected samples

**Fig. 2:**
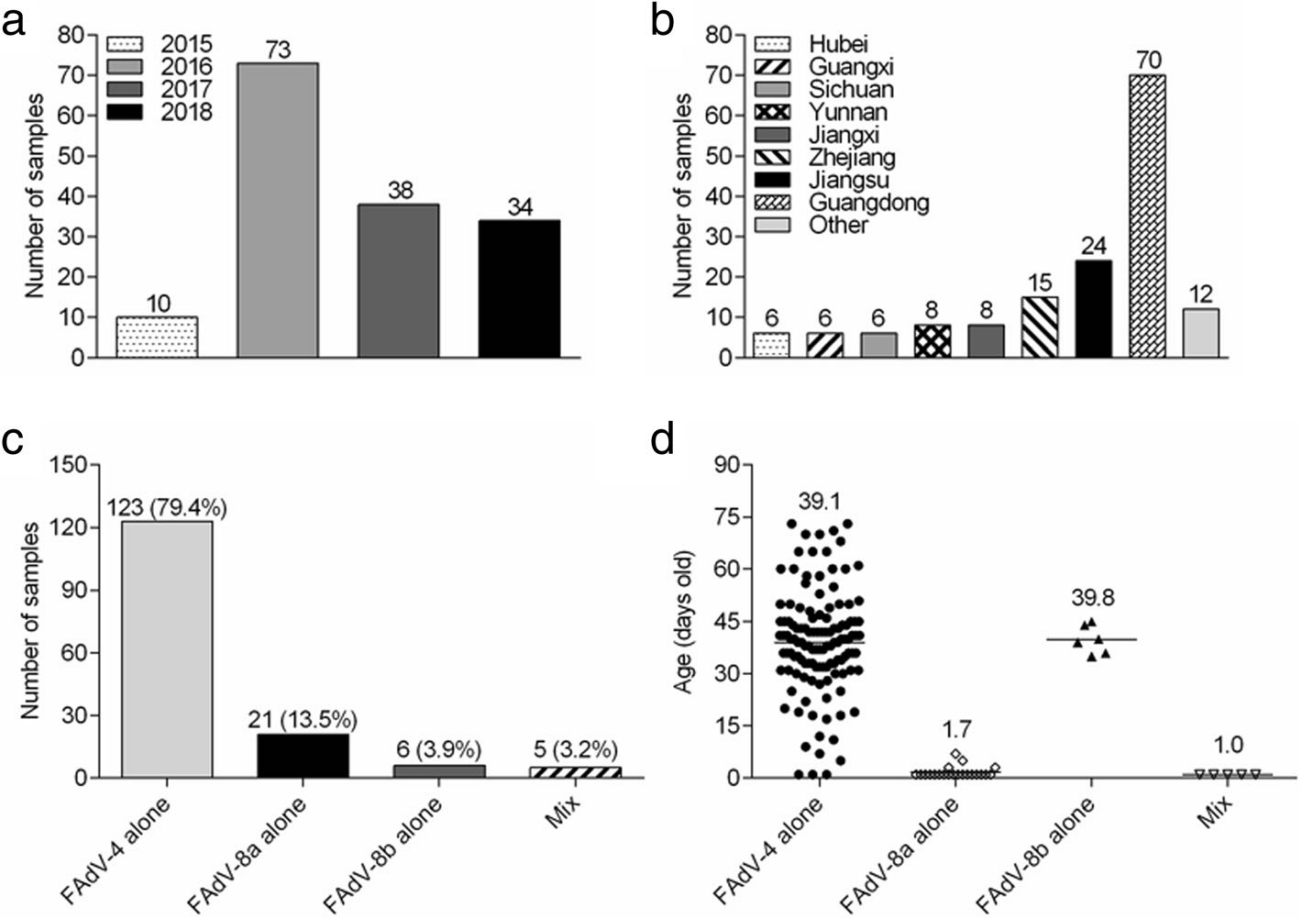
Summary of isolated FAdV strains. (**a**) the distribution of FAdV positive samples from 2015 to 2018, (**b**) the distribution of FAdV positive samples in different regions, (**c**) FAdV serotypes and (**d**) the age of the affected birds and complications

More than 280 suspected clinical cases of IBH or HHS were examined between 2015 and 2018, and these samples were distributed in 15 provinces or cities in China, including Guangdong, Guangxi, Hunan, Hubei, Jiangxi, Sichuan, Chongqing, Yunnan, Zhejiang, Jiangsu, Henan, Shandong, Hebei, Tianjin and Liaoning (Fig. 1). The epidemiological map constructed as a result of current study depicts 280 suspected clinical cases. Among them, 155 samples (55.4%) were positive for FAdV after PCR detection. Temporal analysis showed that the number of positive samples collected from 2015 to 2018 was 10, 73, 38 and 34, respectively, and most of the positive samples were isolated in 2016 (Fig. 2a). Spatial analysis showed that FAdV strains were collected from 15 provinces or cities: 10 provinces were in southern China, and the others were in northern China. In total, 70 strains were isolated in Guangdong Province (Fig. 2b).

### Serotyping of FAdV isolates

*Hexon* gene was sequenced for all 155 positive samples with Sanger method. In 155 cases, 123 (79.4%, 123/155) of the isolates were related to FAdV-C, and 27 (17.4%, 27/155) were identified as FAdV-E, whereas 5 (3.2%, 5/155) of the cases were proven to include two different FAdV strains (FAdV-C with FAdV-E) (Fig. 2c). Phylogenetic analysis based on the *hexon* gene showed that these isolates were clustered into three distinct serotypes: FAdV-4 (79.4%, 123/155), FAdV-8a (13.5%, 21/155) and FAdV-8b (3.9%, 6/155), of which FAdV-4 was the dominant serotype in China. Clinical cases of FAdV-4, FAdV-8a and FAdV-8b infections happened in chickens that were 1–73 days old (average 39.1 days), 1–7 days old (average 1.7 days) and 35–45 days old (average 39.8 days) respectively, and mix infections occurred in 1-day-old chicken (Fig. 2d).

### Phylogenetic analysis of FAdV isolates

**Fig. 3:**
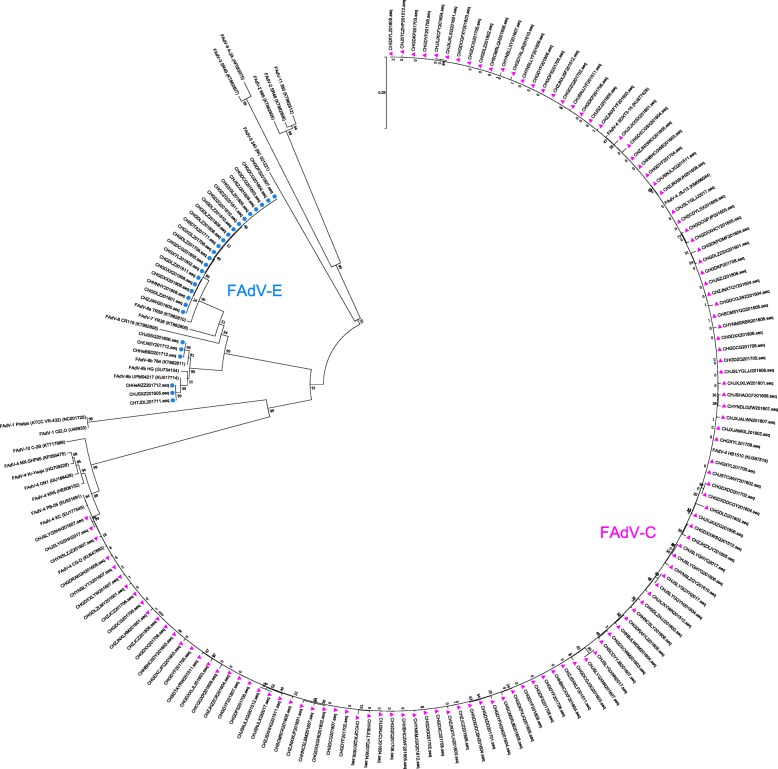
Phylogenetic tree based on *hexon* gene sequences of 155 field strains and other representative FAdV strains. The tree was constructed using MEGA 7.0 software by the neighbor-joining method (1000 replicates for bootstrap).‘’ indicate FAdV-C *hexon* gene sequences and‘‘indicate FAdV-E *hexon* gene sequences

The results of the phylogenetic analysis of FAdV isolated strains and reference strains are shown in Fig. 3. The FAdV strains clustered into two major groups. By computing the pairwise distance of full-length hexon coding sequence, 123 strains from species FAdV-C had 98.7–100% similarities with FAdV-4 reference strains, 21 FAdV-E strains were found to share 99.3–100% similarities with the FAdV-8a reference strains, and six FAdV-E strains possessed 98.3–99.5% identities with FAdV-8b reference strains at the nucleotide level.

## Discussion

Recently, more and more clinical cases of FAdV infection have been reported worldwide, and multiple FAdV strains have been isolated from dead or sick animals [7–23]. In addition, epidemics with mixed serotypes have been observed in different regions, for instance, FAdV-4 and -8 in Asia [10, 18, 23, 24], FAdV-2 and -8b in South Africa [25, 26], FAdV-2, -11, -7 and -8 in Europe and North America [6, 27, 28]. FAdV infections are associated with some avian diseases, such as IBH, HHS and GEU. In China, the prevalence of IBH is sporadic in chickens without resulting in a high mortality rate. However, starting from 2015, outbreaks of HHS have been reported with high mortality rates in several broiler-producing provinces in China due to new FAdV variants. Subsequently, the disease spread throughout the country and lead to tremendous economic losses to the fast-developing poultry industry [18]. FAdV *hexon* gene plays an important part in the molecular epidemiology and in the genetic variation of field strains. In order to prevent and control FAdV infection, it is necessary for us to further investigate the prevalence of FAdV and the molecular characteristics of the *hexon* genes of Chinese FAdV field strains from 2015 to 2018. In this study, the genetic epidemiology of 155 FAdV samples collected between 2015 and 2018 in China was investigated.

It was previously reported that IBH and HHS mainly affected 3–5-week-old chickens, although outbreaks have occasionally occurred among layers [29, 30]. The present epidemiological survey showed that some clinical cases of FAdV infections have affected various ages of chickens and it might be caused by both horizontal and vertical transmission, although we were unable to know whether the route of transmission was horizontal or vertical in each case [18]. It also showed that FAdV infections were associated with different FAdV species in China, and at least two species of FAdVs (species C and E) were detected. In our surveillance of FAdV, IBH outbreaks have been related to FAdV-8a and FAdV-8b, and HHS cases have been related to FAdV-4. In addition, 79.4% FAdV strains were genetically related to serotype 4, proving that this serotype was dominant in China between 2015 and 2018. In a previous epidemiological survey, FAdV-11 was the predominant serotype in some regions of China from 2007 to 2014 [31]. Thus, it is necessary for us to further investigate the prevalence of FAdV, and understand the genetic epidemiology of viruses associated with IBH and HHS.

In our survey, five cases were detected with mixed infections of FAdV strains (FAdV-C with FAdV-E), which confirmed that mixed infections with different FAdV serotypes could happen in the same bird as previously reported [18, 27]. The possible reason for this was that commercial chickens often transmitted vertically and horizontally by fecal route. In this case, multiple infections might occur in a chicken flock due to the reactivation of latent infection virus. So far, it is ambiguous whether mixed infections of adenovirus could aggravate the severity of the clinical diseases [18]. In fact, the control and prevention of disease often centered on the primary epidemic viral serotypes and ignored previous mixed infection of FAdV, this may result in outbreaks of other serotypes in China. Thus, mixed infection of FAdV with multiple serotypes will be a big challenge to prevent and control the disease.

## Conclusions

Fowl adenoviruses (FAdVs) have a worldwide distribution and are associated with a variety of diseases, causing huge economic losses to the poultry industry. In this study, we isolated 155 FAdV strains from diseased chickens from poultry in China between 2015 and 2018, and investigated the molecular characteristics by performing phylogenetic analyses based on the *hexon* genes. It showed that these isolates were clustered into three distinct serotypes: FAdV-4 (79.4%, 123/155), FAdV-8a (13.5%, 21/155) and FAdV-8b (3.9%, 6/155), of which FAdV-4 was the dominant serotype in China. Taken together, these results provided us a clear landscape of genetic epidemiology of FAdV circulating in China during 2015–2018 and would help us for the FAdV prevention.

## Additional file

Additional file 1: **Table S1.** Epidemiological findings of flocks that were positive for FAdV. (DOC 404 kb)

## Data Availability

The datasets used and analyzed during the current study are available from the corresponding author on reasonable request.

## Abbreviations

FAdV-4: Serotype 4 fowl adenovirus
FAdV-8a: Serotype 8a fowl adenovirus
FAdV-8b: Serotype 8b fowl adenovirus
FAdVs: Fowl adenovirus
GEU: Gizzard erosion ulceration
HHS: Hepatitis-hydropericardium syndrome
IBH: Inclusion body hepatitis
PCR: Polymerase chain reaction
SPF: Specific pathogen free
