# Supplementary material for: Epidemiological investigation of fowl adenovirus infections in poultry in China during 2015–2018
Source: BMC Vet Res. 2019 Aug 1;15:271. doi: 10.1186/s12917-019-1969-7 (PMC6676587; doi:10.1186/s12917-019-1969-7)
Supplement: Supplementary file 1 — Table S1. Epidemiological findings of flocks that were positive for FAdV. (DOC 404 kb) [file 12917_2019_1969_MOESM1_ESM.doc]

**Additional file 1:**

**Table S1**. Epidemiological findings of flocks that were positive for FAdV.

| Number | Isolate | Date | Province | Age (day) | Serotype |
| --- | --- | --- | --- | --- | --- |
| 1 | CH/GDXXWSQ/2015 | 2015 | Guangdong | 32 | FAdV-4 |
| 2 | CH/JXJXYWN/2015 | 2015 | Jiangxi | 47 | FAdV-4 |
| 3 | CH/JSNJLXQ/2015 | 2015 | Jiangsu | 43 | FAdV-4 |
| 4 | CH/JSNJJYF/2015 | 2015 | Jiangsu | 39 | FAdV-4 |
| 5 | CH/JSTCZHP/2015 | 2015 | Jiangsu | 42 | FAdV-4 |
| 6 | CH/JSNJLXQI/2015 | 2015 | Jiangsu | 37 | FAdV-4 |
| 7 | CH/SDTAYRW/2015 | 2015 | Shandong | 40 | FAdV-4 |
| 8 | CH/ZJSXHHG/2015 | 2015 | Zhejiang | 32 | FAdV-4 |
| 9 | CH/ZJSXKLF/2015 | 2015 | Zhejiang | 45 | FAdV-4 |
| 10 | CH/JZNXJSF/2015 | 2015 | Zhejiang | 33 | FAdV-4 |
| 11 | CH/CQFXQ/2016 | 2016 | Chongqing | 31 | FAdV-4 |
| 12 | CH/CQQXR/2016 | 2016 | Chongqing | 50 | FAdV-4 |
| 13 | CH/GDCGFXY/2016 | 2016 | Guangdong | 28 | FAdV-4 |
| 14 | CH/GDCGJGQ/2016 | 2016 | Guangdong | 55 | FAdV-4 |
| 15 | CH/GDCGJWZ/2016 | 2016 | Guangdong | 37 | FAdV-4 |
| 16 | CH/GDCGPJP/2016 | 2016 | Guangdong | 40 | FAdV-4 |
| 17 | CH/GDKPGMF/2016 | 2016 | Guangdong | 41 | FAdV-4 |
| 18 | CH/GDKPXJQ/2016 | 2016 | Guangdong | 73 | FAdV-4 |
| 19 | CH/GDLD/2016 | 2016 | Guangdong | 70 | FAdV-4 |
| 20 | CH/GDLZHJJ/2016 | 2016 | Guangdong | 30 | FAdV-4 |
| 21 | CH/GDLZLMY/2016 | 2016 | Guangdong | 31 | FAdV-4 |
| 22 | CH/GDLZZSX2016 | 2016 | Guangdong | 38 | FAdV-4 |
| 23 | CH/GDNXFYF/2016 | 2016 | Guangdong | 50 | FAdV-4 |
| 24 | CH/GDRAFXJ/2016 | 2016 | Guangdong | 31 | FAdV-4 |
| 25 | CH/GDRAKGH/2016 | 2016 | Guangdong | 65 | FAdV-4 |
| 26 | CH/GDXCGSX/2016 | 2016 | Guangdong | 33 | FAdV-4 |
| 27 | CH/GDXCJFQ/2016 | 2016 | Guangdong | 43 | FAdV-4 |
| 28 | CH/GDXCWM/2016 | 2016 | Guangdong | 32 | FAdV-4 |
| 29 | CH/GDXXGHK/2016 | 2016 | Guangdong | 36 | FAdV-4 |
| 30 | CH/GDXXLJL/2016 | 2016 | Guangdong | 42 | FAdV-4 |
| 31 | CH/GDXXLYW/2016 | 2016 | Guangdong | 32 | FAdV-4 |
| 32 | CH/GDXXHCY/2016 | 2016 | Guangdong | 37 | FAdV-4 |
| 33 | CH/GDXDDCGY/2016 | 2016 | Guangdong | 45 | FAdV-4 |
| 34 | CH/GDXDDCBH/2016 | 2016 | Guangdong | 50 | FAdV-4 |
| 35 | CH/GDYALJR/2016 | 2016 | Guangdong | 48 | FAdV-4 |
| 36 | CH/GDYFHGW/2016 | 2016 | Guangdong | 35 | FAdV-4 |
| 37 | CH/HBHCSDY/2016 | 2016 | Hubei | 42 | FAdV-4 |
| 38 | CH/HBHCGWB/2016 | 2016 | Hubei | 33 | FAdV-4 |
| 39 | CH/HBHCXXF/2016 | 2016 | Hubei | 60 | FAdV-4 |
| 40 | CH/HBJLWDM/2016 | 2016 | Hubei | 36 | FAdV-4 |
| 41 | CH/HBJLLYQ/2016 | 2016 | Hubei | 36 | FAdV-4 |
| 42 | CH/HBHCZWF/2016 | 2016 | Hubei | 39 | FAdV-4 |
| 43 | CH/HNCSLY/2016 | 2016 | Hunan | 34 | FAdV-4 |
| 44 | CH/HNCSLBM/2016 | 2016 | Hunan | 22 | FAdV-4 |
| 45 | CH/JSTCWHY/2016 | 2016 | Jiangsu | 50 | FAdV-4 |
| 46 | CH/JSLYGQYH/2016 | 2016 | Jiangsu | 43 | FAdV-4 |
| 47 | CH/JSNJCL/2016 | 2016 | Jiangsu | 44 | FAdV-4 |
| 48 | CH/JSHAOCF/2016 | 2016 | Jiangsu | 46 | FAdV-4 |
| 49 | CH/JSLYGHYQ/2016 | 2016 | Jiangsu | 40 | FAdV-4 |
| 50 | CH/JSLYGLJJ/2016 | 2016 | Jiangsu | 35 | FAdV-4 |
| 51 | CH/JSLYGSHH/2016 | 2016 | Jiangsu | 42 | FAdV-4 |
| 52 | CH/JSLYGXNN/2016 | 2016 | Jiangsu | 33 | FAdV-4 |
| 53 | CH/JSXZ/2016 | 2016 | Jiangsu | 44 | FAdV-8b |
| 54 | CH/JSSQ/2016 | 2016 | Jiangsu | 35 | FAdV-8b |
| 55 | CH/JXJXXSX/2016 | 2016 | Jiangxi | 45 | FAdV-4 |
| 56 | CH/JXJXLW/2016 | 2016 | Jiangxi | 40 | FAdV-4 |
| 57 | CH/JXJXLSG/2016 | 2016 | Jiangxi | 39 | FAdV-4 |
| 58 | CH/JXJAMGL/2016 | 2016 | Jiangxi | 44 | FAdV-4 |
| 59 | CH/JXJXCFY/2016 | 2016 | Jiangxi | 45 | FAdV-4 |
| 60 | CH/JXJAXZG/2016 | 2016 | Jiangxi | 34 | FAdV-4 |
| 61 | CH/JXJALWN/2016 | 2016 | Jiangxi | 30 | FAdV-4 |
| 62 | CH/SCMSRBR/2016 | 2016 | Sichuan | 36 | FAdV-4 |
| 63 | CH/SCMSCK/2016 | 2016 | Sichuan | 51 | FAdV-4 |
| 64 | CH/SCMSLQH/2016 | 2016 | Sichuan | 73 | FAdV-4 |
| 65 | CH/SCDYYJB/2016 | 2016 | Sichuan | 42 | FAdV-4 |
| 66 | CH/SCMSYQQ/2016 | 2016 | Sichuan | 45 | FAdV-4 |
| 67 | CH/SCMSRJB/2016 | 2016 | Sichuan | 41 | FAdV-4 |
| 68 | CH/SCDYLSX/2016 | 2016 | Sichuan | 43 | FAdV-4 |
| 69 | CH/YNSLYYX/2016 | 2016 | Yunnan | 65 | FAdV-4 |
| 70 | CH/YNSLZJZ/2016 | 2016 | Yunnan | 58 | FAdV-4 |
| 71 | CH/YNSLLXY/2016 | 2016 | Yunnan | 40 | FAdV-4 |
| 72 | CH/YNDLGZW/2016 | 2016 | Yunnan | 60 | FAdV-4 |
| 73 | CH/YNSLLYF/2016 | 2016 | Yunnan | 61 | FAdV-4 |
| 74 | CH/YNSLZGY/2016 | 2016 | Yunnan | 60 | FAdV-4 |
| 75 | CH/YNKMJGX/2016 | 2016 | Yunnan | 49 | FAdV-4 |
| 76 | CH/ZJRAWJH/2016 | 2016 | Zhejiang | 25 | FAdV-4 |
| 77 | CH/ZJNXLHM/2016 | 2016 | Zhejiang | 53 | FAdV-4 |
| 78 | CH/ZJNXWJF/2016 | 2016 | Zhejiang | 41 | FAdV-4 |
| 79 | CH/ZJNXYLH/2016 | 2016 | Zhejiang | 46 | FAdV-4 |
| 80 | CH/ZJNXTQY/2016 | 2016 | Zhejiang | 65 | FAdV-4 |
| 81 | CH/ZJHZZCR/2016 | 2016 | Zhejiang | 37 | FAdV-4 |
| 82 | CH/ZJHZXJY/2016 | 2016 | Zhejiang | 36 | FAdV-4 |
| 83 | CH/ZJNXWKX/2016 | 2016 | Zhejiang | 58 | FAdV-4 |
| 84 | CH/GDXX/201706 | 2017 | Guangdong | 43 | FAdV-4 |
| 85 | CH/GDYF/201706 | 2017 | Guangdong | 35 | FAdV-4 |
| 86 | CH/GDZQ/201702 | 2017 | Guangdong | 18 | FAdV-4 |
| 87 | CH/GDZQ/201706 | 2017 | Guangdong | 68 | FAdV-4 |
| 88 | CH/GDCG/201708 | 2017 | Guangdong | 25 | FAdV-4 |
| 89 | CH/GDFS/201708 | 2017 | Guangdong | 19 | FAdV-4 |
| 90 | CH/GDXC/201708 | 2017 | Guangdong | 39 | FAdV-4 |
| 91 | CH/GDYF/201708 | 2017 | Guangdong | 5 | FAdV-4 |
| 92 | CH/GDCG/201705 | 2017 | Guangdong | 41 | FAdV-4 |
| 93 | CH/GDKP/201703 | 2017 | Guangdong | 28 | FAdV-4 |
| 94 | CH/GDKP/201708 | 2017 | Guangdong | 17 | FAdV-4 |
| 95 | CH/GDYF/201702 | 2017 | Guangdong | 38 | FAdV-4 |
| 96 | CH/GDYF/201705 | 2017 | Guangdong | 42 | FAdV-4 |
| 97 | CH/GDCG/201706 | 2017 | Guangdong | 38 | FAdV-4 |
| 98 | CH/GDFS/201706 | 2017 | Guangdong | 56 | FAdV-4 |
| 99 | CH/GDFS/201705 | 2017 | Guangdong | 49 | FAdV-4 |
| 100 | CH/GDYF/201704 | 2017 | Guangdong | 30 | FAdV-4 |
| 101 | CH/GDKP/201706 | 2017 | Guangdong | 33 | FAdV-4 |
| 102 | CH/GDXX/201705 | 2017 | Guangdong | 44 | FAdV-4 |
| 103 | CH/GDZQ/201705 | 2017 | Guangdong | 11 | FAdV-4 |
| 104 | CH/GDXDDCBH/2017 | 2017 | Guangdong | 71 | FAdV-4 |
| 105 | CH/GDXDDCGY/2017 | 2017 | Guangdong | 9 | FAdV-4 |
| 106 | CH/GXYL/201705 | 2017 | Guangxi | 19 | FAdV-4 |
| 107 | CH/GXYL/201706 | 2017 | Guangxi | 1 | FAdV-4 |
| 108 | CH/JSLYGHYQ/2017 | 2017 | Jiangsu | 31 | FAdV-4 |
| 109 | CH/JSLYGLJJ/2017 | 2017 | Jiangsu | 45 | FAdV-4 |
| 110 | CH/JSLYGQYH/2017 | 2017 | Jiangsu | 41 | FAdV-4 |
| 111 | CH/JSLYGSHH/2017 | 2017 | Jiangsu | 38 | FAdV-4 |
| 112 | CH/JSLYGXNN/2017 | 2017 | Jiangsu | 60 | FAdV-4 |
| 113 | CH/JSNJLXQI/2017 | 2017 | Jiangsu | 70 | FAdV-4 |
| 114 | CH/ZJCZ/201706 | 2017 | Zhejiang | 1 | FAdV-4 |
| 115 | CH/GDLZ/201708 | 2017 | Guangdong | 1 | FAdV-8a |
| 116 | CH/GXGL/201708 | 2017 | Guangxi | 1 | FAdV-8a |
| 117 | CH/SDTA/201711 | 2017 | Shandong | 7 | FAdV-8a |
| 118 | CH/HeBBD/201712 | 2017 | Hebei | 39 | FAdV-8b |
| 119 | CH/HeNZZ/201712 | 2017 | Henan | 45 | FAdV-8b |
| 120 | CH/LNSY/201712 | 2017 | Liaoning | 36 | FAdV-8b |
| 121 | CH/TJDL/201711 | 2017 | Tianjin | 40 | FAdV-8b |
| 122 | CH/GDCG/201807 | 2018 | Guangdong | 23 | FAdV-4 |
| 123 | CH/GDLZ/201802 | 2018 | Guangdong | 18 | FAdV-4 |
| 124 | CH/GDXX/201806 | 2018 | Guangdong | 41 | FAdV-4 |
| 125 | CH/GDYF/201806 | 2018 | Guangdong | 27 | FAdV-4 |
| 126 | CH/GDYF/201807 | 2018 | Guangdong | 7 | FAdV-4 |
| 127 | CH/GDYF/201808 | 2018 | Guangdong | 36 | FAdV-4 |
| 128 | CH/GXYL/201805 | 2018 | Guangxi | 34 | FAdV-4 |
| 129 | CH/JSZJ/201805 | 2018 | Jiangsu | 20 | FAdV-4 |
| 130 | CH/JSZJ/201808 | 2018 | Jiangsu | 12 | FAdV-4 |
| 131 | CH/ZJCZ/201806 | 2018 | Zhejiang | 1 | FAdV-4 |
| 132 | CH/ZJCZ/201808 | 2018 | Zhejiang | 29 | FAdV-4 |
| 133 | CH/GDCG/201803 | 2018 | Guangdong | 1 | FAdV-8a |
| 134 | CH/GDCG/201804 | 2018 | Guangdong | 1 | FAdV-8a |
| 135 | CH/GDCG/201805 | 2018 | Guangdong | 1 | FAdV-8a |
| 136 | CH/GDFS/201807 | 2018 | Guangdong | 1 | FAdV-8a |
| 137 | CH/GDLZ/201801 | 2018 | Guangdong | 3 | FAdV-8a |
| 138 | CH/GDLZ/201806 | 2018 | Guangdong | 1 | FAdV-8a |
| 139 | CH/GDLZ/201808 | 2018 | Guangdong | 1 | FAdV-8a |
| 140 | CH/GDLZ/201810 | 2018 | Guangdong | 5 | FAdV-8a |
| 141 | CH/GDLZ/201811 | 2018 | Guangdong | 1 | FAdV-8a |
| 142 | CH/GDXX/201808 | 2018 | Guangdong | 1 | FAdV-8a |
| 143 | CH/GDXX/201809 | 2018 | Guangdong | 3 | FAdV-8a |
| 144 | CH/GDZQ/201810 | 2018 | Guangdong | 1 | FAdV-8a |
| 145 | CH/GDZQ/201811 | 2018 | Guangdong | 1 | FAdV-8a |
| 146 | CH/GXGL/201805 | 2018 | Guangxi | 1 | FAdV-8a |
| 147 | CH/GXYL/201802 | 2018 | Guangxi | 1 | FAdV-8a |
| 148 | CH/HNHY/201808 | 2018 | Hunan | 1 | FAdV-8a |
| 149 | CH/JSZJ/201809 | 2018 | Jiangsu | 1 | FAdV-8a |
| 150 | CH/ZJWH/201805 | 2018 | Zhejiang | 1 | FAdV-8a |
| 151 | CH/GDLZXX/201809 | 2018 | Guangdong | 1 | FAdV-8a/FAdV-4 |
| 152 | CH/GDYFCG/201807 | 2018 | Guangdong | 1 | FAdV-8a/FAdV-4 |
| 153 | CH/GDYFCG/201810 | 2018 | Guangdong | 1 | FAdV-8a/FAdV-4 |
| 154 | CH/HNHY/201810 | 2018 | Hunan | 1 | FAdV-8a/FAdV-4 |
| 155 | CH/JSZJ/201808 | 2018 | Jiangsu | 1 | FAdV-8a/FAdV-4 |
